# Can telomere length predict bone health? A review of current evidence

**DOI:** 10.17305/bjbms.2020.4664

**Published:** 2020-11

**Authors:** Sok Kuan Wong, Soelaiman Ima-Nirwana, Kok-Yong Chin

**Affiliations:** 1 Department of Pharmacology, Faculty of Medicine, Universiti Kebangsaan Malaysia, Cheras, Kuala Lumpur, Malaysia; 2 State Key Laboratory of Oncogenes and Related Genes, Renji-Med X Clinical Stem Cell Research Center, Department of Urology, Ren Ji Hospital, School of Medicine, Shanghai Jiao Tong University, Shanghai, China

**Keywords:** Bone health, osteoblast, osteoclast, osteoporosis, telomerase, telomere

## Abstract

Telomeres are repetitive DNA sequences located at the end of chromosomes that serve as a protective barrier against chromosomal deterioration during cell division. Approximately 50–200 base pairs of nucleotides are lost per cell division, and new repetitive nucleotides are added by the enzyme telomerase, allowing telomere maintenance. Telomere shortening has been proposed as an indicator for biological aging, but its relationship with age-related osteoporosis is ambiguous. We summarize the current evidence on the relationship between telomere length and bone health in experimental and epidemiological studies, which serve as a scientific reference for the development of novel diagnostic markers of osteoporosis or novel therapeutics targeting telomere and telomerase of bone cells to treat osteoporosis.

## INTRODUCTION

Osteoporosis is a degenerative bone disorder ­characterized by deterioration of bone mass, microstructure and mechanical strength, leading to an increased tendency of fragility fracture [1,2]. Osteoporosis has emerged as a public health issue in the aging society. Osteoporosis imposes an enormous burden at the national level, in terms of healthcare cost and loss of productivity, as well as at the individual level, in terms of the direct and indirect medical cost of treating the fracture and the reduced quality of life of the patients [3]. Age-related decline of sex hormones is the main cause of primary osteoporosis by causing an increase in bone resorption by osteoclasts and reduced bone formation by osteoblasts, leading to bone loss [4]. Current osteoporosis prevention emphasizes the importance of attaining optimal peak bone mass during childhood and adolescence, avoiding premature bone loss during adulthood, and preventing falls and fractures in old age through lifestyle, dietary, and pharmacological interventions [5,6].

Telomeres are short repeats of highly conserved nucleotides with a TTAGGG/CCCTAA sequence at the ends of chromosomes, preventing the deterioration of double-strand free ends to ensure chromosomal stability and integrity [7]. Telomere length indicative of cellular senescence has been regarded as a marker for the progression of age-related diseases [8]. Telomeres shorten with every round of cell division to prevent further division and induce cellular aging; this process occurs in most human tissues, including fibroblasts [9], lymphocytes [10], keratinocytes [11] and chondrocytes [12]. Previous studies also indicated that telomere shortening limits the proliferation, differentiation, and normal function of bone cells [13,14]. Telomere attrition can be reversed by telomere maintenance that requires the enzyme telomerase to compensate and elongate telomeric sequences, thereby prolonging the lifespan of cells [15]. In this regard, telomere shortening and/or telomerase deficiency may be considered as a potential biomarker for the diagnosis of age-related bone loss.

In this review, we summarize the documented literature on the role of telomere and telomerase in maintaining bone health. Evidence from *in vivo*, *in vitro*, and human studies was collated. This review provides a scientific overview of the mechanism of how telomere shortening and deficiency affect bone health, which could serve as disease markers or drug targets for the development of therapeutic agents for osteoporosis.

## RELATIONSHIP BETWEEN TELOMERE LENGTH AND BONE HEALTH: EVIDENCE FROM *IN VITRO* STUDIES

Several *in vitro* studies on the effects of telomere length on bone cells are available (Table 1). Using human trabecular osteoblasts (designated as K73) obtained from a 73-year-old healthy woman, Kveiborg et al. (1999) measured the telomere restriction fragment (TRF) length in cells undergoing cellular aging. The β-galactosidase activity, as an indicator of cellular senescence, was revealed by immunofluorescence. In this study, less than 10% of the cells exhibited β-galactosidase activity in the young culture, whereas more than 95% of cells exhibited β-galactosidase activity in the old culture. The TRF length in human trabecular osteoblasts was also measured in relation to their *in vitro* age. The results showed that the TRF length was 9.32 kb in middle-aged cells and 7.80 kb in old cells. On average, the rate of telomere shortening was approximately 100 bp per population doubling [16].

**TABLE 1:**
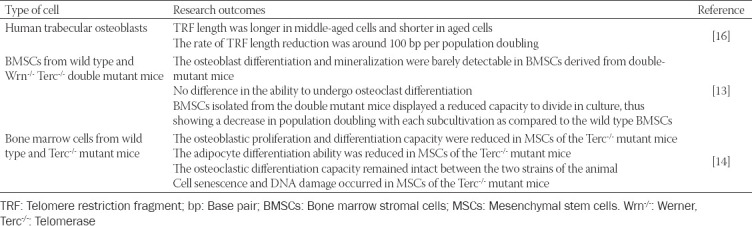
The effects of telomere length on bone cells *in vitro*

The effects of telomere shortening on the formation of bone cells have also been studied. Bone marrow stromal cells (BMSCs) and bone marrow-derived macrophages from wild type and Werner (Wrn^-/-^) telomerase (Terc^-/-^) double-mutant mice, which suffer from premature aging, were cultured to demonstrate the association between senescence and bone cell differentiation that result in aged-related osteoporosis. Impaired osteoblast differentiation and mineralization, as shown by reduced alkaline phosphatase (ALP) activity, calcium nodule formation, osteopontin and osteocalcin expressions and intact osteoclast differentiation, indicated by unchanged tartrate-resistant acid phosphatase (TRAP)-positive cells were observed in BMSCs derived from these mice. The cells also exhibited accelerated aging, as shown by a reduction in cell division capacity in culture [13].

In a more comprehensive study, Saeed et al. studied the cellular mechanism of bone loss *in vitro* using bone marrow mesenchymal stem cells (MSCs) and bone marrow cells derived from wild type and Terc^-/-^ mice. In this study, the authors revealed that the osteoblastic differentiation capacity was impaired in MSCs from Terc^-/-^ mice. The number of osteogenic colonies, proliferating cells, the ALP activity and the expression of osteoblast-specific genes, such as runt-related transcription factor-2 (*RUNX2*), osterix (*SP7*), tissue non-specific alkaline phosphatase (*ALPL*), collagen type 1 alpha 1 (*COL1A1*), osteopontin (*SPP1*), integrin-binding sialoprotein (*IBSP*), osteonectin/secreted protein acidic cysteine-rich (*SPARC*), and osteocalcin (*BGLAP*) were reduced. In addition to the impairment of osteoblastogenesis in MSCs from Terc^-/-^ mice, defective adipogenesis was also observed, as indicated by a decreased number of lipid-filled mature adipocytes and expression of adipocyte-specific markers including peroxisome proliferator-activator receptor-gamma (PPAR-γ), adiponectin (AdipoQ), and adipocyte protein 2 (aP2). There was no significant difference in TRAP staining of multinucleated cells between the two strains of animal, indicating that the Terc^-/-^ mice osteoclastic cells exhibited a normal phenotype. Moreover, a larger proportion of β-galactosidase-positive cells and gamma-H2A histone family member X-positive (γ-H2AX^+^) cells were detected in Terc^-/-^ MSCs, which are indicators of cell aging and DNA damage, respectively [14].

Overall, the currently available preclinical evidence recapitulates the possible link between impaired bone health and shortening and/or dysfunctional telomeres. These studies strongly suggest that age-related telomere dysfunction mainly affects the bone-forming osteoblasts adversely, but not the bone-resorbing osteoclasts, resulting in the uncoupling of the bone remodeling process to favor bone loss and the development of osteoporosis. It is speculated that osteocytes may also be affected since they are the terminally differentiated osteoblasts, but there has been no study on this aspect. Although the evidence is insufficient, it is reasonable to consider the causal role of telomere attrition and/or telomere uncapping in the impairment of bone homeostasis.

## RELATIONSHIP BETWEEN TELOMERE LENGTH AND BONE HEALTH: EVIDENCE FROM *IN VIVO* STUDIES

The investigation on the effects of telomere length and bone health *in vivo* is limited (Table 2). Two strains of male animals (senescence-accelerated mouse resistance 1 [SAMR1] and senescence-accelerated mouse prone 6 [SAMP6] mice) were used to evaluate the bone quality and telomere length in liver tissue. Quantitative micro-computed tomography (micro-CT) analysis confirmed that osteoporosis traits were observed in the trabecular and cortical bones of SAMP6 mice, as evidenced by decreased trabecular bone volume density (tBV/TV), trabecular bone surface density (tBS/TV), trabecular number (Tb.N), apparent volume density, cortical bone volume density (cBV/TV), and increased trabecular separation (Tb.Sp). Lower values for the polar and second moments of inertia were also detected in SAMP6 mice, signifying the decrease in bending stiffness and torsion strength. The shortening of telomere length was also validated using several approaches, including quantitative fluorescence *in situ* hybridization and quantitative Southern blot analysis, supporting the concept of telomere deficiency as a contributor to accelerated aging in SAMP6 mice [17].

**TABLE 2:**
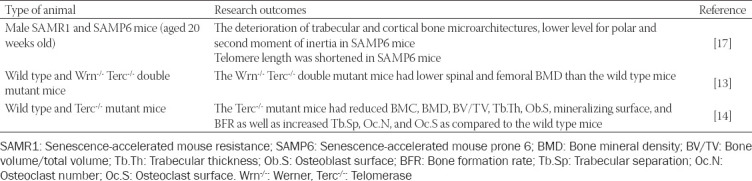
The effects of telomere length on bone in animals

The role of telomerase in the skeleton was also investigated in previous studies. Insufficiency of telomerase expression results in gradual telomere shortening in each cell division, followed by replicative cell senescence or programmed cell death due to the inability of the telomere to protect chromosome ends [18]. Pignolo et al. used a mouse model with mutations in Werner (Wrn^-/-^) and telomerase (Terc^-/-^) genes to explore the impact of defects in telomere maintenance on bone phenotype, assessed by dual-energy X-ray absorptiometry (DXA) and micro-CT. This animal model developed Werner syndrome (an autosomal-recessive genetic disorder characterized by premature aging and an increased risk of other diseases [19]) and telomerase dysfunction. In this study, the Wrn^-/-^ Terc^-/-^ mutant mice exhibited lower spine bone mineral density (BMD) compared to the wild type controls. Detailed micro-CT analysis also confirmed that the trabecular BMD at the distal femur of the Wrn^-/-^ Terc^-/-^ mutant mice was significantly lower than the wild type mice, but comparable findings were not observed in cortical BMD [13]. Another research group also reported that age-related bone loss occurred in telomerase-deficient mice, confirmed by DXA, micro-CT, and bone histomorphometry. Significant reductions in tBV/TV, trabecular thickness (Tb.Th), cortical thickness (Ct.Th), bone mineral content and BMD, and an increase in Tb.Sp compared to the wild type animals were also observed. The bone histomorphometric analysis also showed that the osteoblast surface (Ob.S), mineralizing surface and bone formation rate were reduced, whereas the osteoclast surface (Oc.S) was increased in the Terc^-/-^ mutant mice [14].

## RELATIONSHIP BETWEEN TELOMERE LENGTH AND BONE HEALTH: EVIDENCE FROM HUMAN STUDIES

In an early observational study comprising 110 elderly healthy male subjects (aged 71–86 years), the mean TRF length of peripheral blood leukocytes was inversely correlated with age (*r* = -0.19; *p* = 0.049). The age-corrected mean TRF length correlated positively with the BMD measured at different distal forearm sites, including the mid-region of the forearm (*r* = 0.24; *p* = 0.03), ultradistal forearm (*r* = 0.18; *p* = 0.10), and the total forearm (*r* = 0.22; *p* = 0.04). Subjects with the shortest mean TRF length had the highest bone loss. Subjects with significant bone loss at the mid-region of the forearm had a significant 423 base pairs shorter mean TRF compared to their counterparts without bone loss. Findings from this study suggest that telomere length is predictive of bone loss [20]. A subsequent study by Valdes et al. (2007) involving 2150 female twins aged 18–80 years revealed three important outcomes. Firstly, significant positive correlations were found between the telomere length of leukocytes and BMD at the lumbar spine (*r* = 0.0583; *p* = 0.0050) and forearm (*r* = 0.0539; *p* = 0.0127). Secondly, women with longer telomeres had lower odds of having osteoporosis at two or more sites (odds ratio = 0.594; 95% confidence interval [CI]: 0.42, 0.84). Thirdly, women with osteoporosis had a shorter telomere length (117 base pairs), which is equivalent to 5.2 years older in telomeric age compared to age-matched healthy controls [21].

In a cross-sectional study involving women living with human immunodeficiency virus [HIV] (*n* = 73; aged 43 ± 9 years) and reference controls (*n* = 280; aged 50 ± 8 years), Kalyan et al. (2018) identified that women living with HIV had the greatest deficit in BMD Z-score at the lumbar spine (mean difference = -0.39; 95% CI: -0.61, -0.17) and total hip (mean ­difference = -0.29; 95% CI: -0.52, -0.07). Women living with HIV aged ≥50 years also had lower BMD T-scores at lumbar spine (mean difference = -0.74; 95% CI: -1.34, -0.15) and total hip (mean difference = -0.52; 95% CI: -1.01, -0.02). Among women living with HIV, lumbar spine BMD was associated with leukocyte telomere length (R^2^ = 0.09; *p* = 0.009), indicating that leukocyte telomere length was strongly associated with lower BMD in women living with HIV [22].

Tao et al. (2019) conducted a study among elderly Chinese men (*n* = 433; aged 68.7 ± 6.3 years) and women (*n* = 584; aged 66.4 ± 5.7 years) to examine the association between leukocyte telomere length, BMD, and osteoporosis. They reported that leukocyte telomere length was negatively correlated with age in men (*r* = -0.1202; *p* = 0.0302) and women (*r* = -0.1158; *p* = 0.0055). Multiple linear regression showed that a shorter leukocyte telomere length contributed to a lower BMD at the femoral neck in women <60 years old (β = 0.346; 95% CI: 0.095, 0.598), and this positive association decreased slowly with increasing age. Among women <65 years old, shorter leukocyte telomere length was also associated with the number of skeletal sites with T-score < −2.5 SD (total hip and lumbar spine) and increased risk of osteoporosis (determined at lumbar spine), but the association was lost with increasing age. In contrast, the outcomes observed in women could not be established in men; hence, the possible predictive role of leukocyte telomere length on bone loss differs by sex [23].

On the contrary, several previous studies did not support the notion that individuals with shorter telomere length would have poor bone health. Kveiborg et al. (1999) compared telomere length in peripheral blood leukocytes collected from young (*n* = 15; aged 20–26 years), elderly (*n* = 15; aged 48–85 years), and osteoporotic (*n* = 14; aged 52–81 years) women and noted that TRF length in peripheral blood leukocytes among young (6.76 ± 0.64 kb), elderly (6.42 ± 0.71 kb), and osteoporotic (6.47 ± 0.94 kb) women was not statistically significant. There were also no correlations between TRF length in peripheral blood leukocytes and bone mass at the spine, femoral neck, and total body among the three groups of women. These results suggest that osteoporosis is not a disease of a generalized premature cellular aging [16]. In another study, a total of 2750 community-dwelling septuagenarians from the Health, Aging, and Body Composition (Health ABC) study were enrolled to examine the relationship between telomere length and BMD, osteoporosis, or fracture. Negative associations between telomere length with age, weight, fasting insulin, and fasting glucose were reported in elderly men and women. However, telomere length was not associated with BMD at the total hip or femoral neck, changes in BMD over time, baseline fracture, and the number of incident fractures over 7 years among elderly men and women in this population [24].

Another cross-sectional study that recruited 1867 community-living Chinese elderly aged 65 years revealed that telomere length was not associated with BMD at the total hip and femoral neck in both men and women [25]. Nielsen et al. (2015) conducted a cohort study to investigate whether a shorter leukocyte telomere length could predict lower BMD among 460 women aged between 25 and 93 years and found a strong negative relationship between chronological age and leukocyte telomere length (β = -0.03; 95% CI: -0.005, -0.002). However, there was no statistical significant association between leukocyte telomere length and BMD at any of the measured sites including the lumbar spine (β = -0.10; 95% CI: -0.71, 0.52), right total hip (β = -0.13; 95% CI: -0.66, 0.41), left total hip (β = -0.13; 95% CI: -0.67, 0.42), right femoral neck (β = -0.03; 95% CI: -0.58, 0.52), and left femoral neck (β = 0.09; 95% CI: -0.47, 0.66) [26].

Taken together, the outcomes from these human studies appear heterogeneous and inconclusive since four human studies indicated a possible relationship between short telomeres and poor bone health, but the other four studies demonstrated paradoxical findings (Table 3). There are several limitations to these included studies. Some studies were conducted using a small sample size; thus, they were not powered to determine small size effects of the relationship between BMD and telomere length [16,20,22]. Moreover, the populations recruited in some of the studies were very specific (such as elderly men or women or women with HIV), making generalization of the results difficult.

**TABLE 3:**
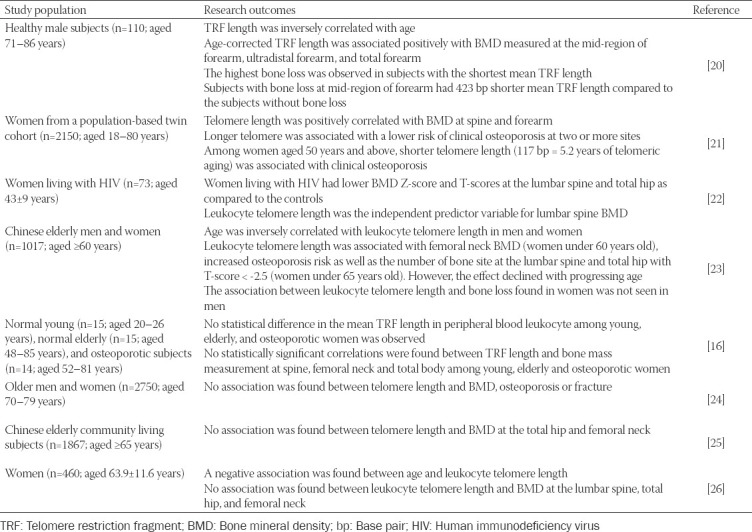
The effects of telomere length on bone in humans

## ROLE OF INFLAMMATION ON TELOMERASE SHORTENING AND OSTEOPOROSIS

Some researchers have postulated that systemic inflammation is the common mechanism underlying the relationship between telomere shortening and osteoporosis [14,21,24]. In humans, telomere length was found to inversely correlate with the serum levels of C-reactive protein (CRP) and interleukin (IL)-6 [21,24]. Furthermore, women with osteoporosis at two or more anatomical sites had higher CRP levels than women without osteoporosis [21]. Overexpression of pro-inflammatory genes involved in osteoclast differentiation was also detected in bone marrow cells from telomerase-deficient mice. Of note, the upregulated genes were IL-1β (*IL1B*), c-Fos proto-oncogene (*FOS*), JunB proto-oncogene (*JUNB*), suppressor of cytokine signaling 3 (*SOCS3*), toll-like receptor 2 (*TLR2*), and tumor necrosis factor-alpha (*TNF*) [14]. The pro-inflammatory microenvironment provides an ideal condition for osteoclastogenesis, mainly mediated through the receptor activator of nuclear factor-kappa B (RANK)/receptor activator of nuclear factor-kappa B ligand (RANKL)/osteoprotegerin (OPG) system [27,28].

## RELATIONSHIP BETWEEN TELOMERE LENGTH AND OTHER RISK FACTORS FOR OSTEOPOROSIS

Other risk factors of osteoporosis include, but are not limited to, sex hormone deficiency, calcium and/or vitamin D deficiency, cigarette smoking, and the use of medications. Intriguingly, these risk factors may impact telomere length. In a study by Lee et al. (2005) involving postmenopausal women, it was found that the relative telomere length in the group on long-term estrogen and progesterone therapy was greater than that in the group who did not receive estrogen and progesterone therapy [29]. With regard to the relationship between plasma calcium level and telomere length, a negative association was reported in South Australian older women [30]. Previous studies also reported that higher plasma 1,25-dihydroxyvitamin D [1,25(OH)_2_D] corresponded to longer telomeres [31] and that vitamin D supplementation significantly increased telomerase activity in peripheral blood mononuclear cells (PBMCs) [32]. Additionally, Verde et al. identified that tobacco consumption caused a reduction in leukocyte telomere length [33]. Furthermore, the use of medications, for instance, glucocorticoids [34] and proton pump inhibitors (PPIs) [35], has been previously shown to induce telomere shortening. Patients with non-functioning pituitary adenomas displaying adrenal insufficiency receiving hydrocortisone replacement have also been shown to have shorter telomeres [34]. Moreover, chronic exposure to esomeprazole impaired endothelial function and accelerated endothelial senescence by reducing telomere length [35]. Taken together, this evidence suggests that telomere shortening could mediate the relationship between these risk factors and the initiation or progression of osteoporosis.

## RELATIONSHIP BETWEEN TELOMERE LENGTH AND OTHER AGING CONDITIONS

The impact of telomere shortening on other age-related diseases, including hypertension, diabetes, cardiovascular diseases, cancer and Alzheimer’s disease, has been previously reviewed [36]. Studies have shown that the relative telomere length in hypertensive patients was significantly shorter than normal subjects in an Indian population [37]. A reverse relationship was also reported between telomere length and cardiovascular diseases. The length of TRF was inversely correlated with aging, and each 1-kb decrease in TRF length was associated with an increased risk of myocardial infarction and stroke [38]. Several studies demonstrated a relationship between telomere length and type 1 [39] and type 2 diabetes mellitus [40], as well as its associated complications [41,42]. In cancer, reduced telomere length was associated with a lower survival rate after cancer [43], but a higher cancer incidence and mortality [44]. However, a similar trend was not observed in Alzheimer’s disease. Lukens et al. (2009) found that telomere length in peripheral blood leukocytes and cerebellum was directly associated with the risk of Alzheimer’s disease. No significant difference in telomere length of the cerebellum was found between the Alzheimer’s disease patients and the aged-matched controls [45].

## CONCLUSION AND FUTURE PERSPECTIVES

Telomere length may be useful for monitoring the biological aging of bone cells. However, the use of telomere length as a biomarker in predicting osteoporosis and fracture remains hypothetical. Several limitations need to be addressed in the current state of knowledge. There is a lack of preclinical studies investigating the effects of telomere length and bone health. The relationship between telomere shortening and the uncoupling of bone remodeling processes found in preclinical data has not always been consistent with human observational studies for a variety of reasons. Considering that bone cells are not easily accessible in clinical settings, the measurement of the telomere length of different cell types, for instance, peripheral blood leukocytes rather than osteoblasts, might cause discrepancies. In addition, differences in the term of the study design, study population, and sample size could have resulted in heterogeneous findings. Moreover, osteoporosis is a multifactorial and complex medical condition, in which pathophysiological mechanisms other than age-dependent telomere shortening may play a larger role.

To improve the shortcomings in the studies outlined, further investigations in a controlled environment using preclinical models are warranted. Animals of old age could be used to validate the relationship between telomere length variation and osteoporosis. There is also a significant research gap to be filled by investigators, particularly on the longitudinal assessment of the effects of telomere length on bone loss in humans. If validated, telomere length can be used as a predictor for bone loss, allowing early steps to be taken to prevent bone loss. It may also provide novel therapeutic avenues for osteoporosis treatment if targeted modulation of telomere length in bone cells can be achieved.
